# Biophysical characterization and ion transport with cell‐based and proteoliposome reconstitution assays of invertebrate K^+^‐Cl^−^ cotransporters

**DOI:** 10.1002/2211-5463.70063

**Published:** 2025-06-13

**Authors:** Satoshi Fudo, Marina Verkhovskaya, Coralie Di Scala, Claudio Rivera, Tommi Kajander

**Affiliations:** ^1^ Institute of Biotechnology, HiLIFE University of Helsinki Finland; ^2^ Neuroscience Center, HiLIFE University of Helsinki Finland; ^3^ INSERM, INMED Aix Marseille University France

**Keywords:** biophysical characterization, cation/chloride cotransporters, ion transport assay, KCC, membrane protein

## Abstract

The cation‐chloride cotransporter (CCC) family includes ion symporters that cotransport monovalent cations and Cl^−^, playing a crucial role in controlling cytoplasmic ion content. K^+^‐Cl^−^ cotransporters (KCCs) facilitate the symport of ions across the plasma membrane. The CCCs participate in various physiological processes, such as transepithelial ion transport and regulation of cell volume. Among KCCs, KCC2 has unique and essential functions in the central nervous system. KCC from *Drosophila melanogaster* (*Dm*KCC) is an ortholog of mammalian KCCs. Its critical role in neuronal transmission has been demonstrated. Also, the cnidarian *Hydra vulgaris* has a functional KCC (*Hv*KCC). Comparative analyses of these transporters with vertebrate counterparts can provide insights into the mechanism of KCC ion transport, regulation, and evolution. Thus, here we purified *Dm*KCC and *Hv*KCC and characterized their biophysical properties using differential scanning fluorimetry and light scattering. We evaluated their functionality in cells and developed a method to study ion transport with flame photometry. Further, a fluorescence‐based assay for *Dm*KCC reconstituted into proteoliposomes was developed. The activity of *Dm*KCC was found to be dependent on Ca^2+^, which is reminiscent of some other chloride transport protein families and potentially important for the KCC protein family overall.

## Graphical abstract

The K^+^‐Cl^−^ cotransporters (KCCs) facilitate the symport of ions across the plasma membrane. They participate in physiological processes including neuronal regulation. Here, we characterized KCCs from *Drosophila* and *Hydra vulgaris*. Comparative analyses of transporters provide insights into the mechanism of KCC ion transport, regulation, and evolution. Thus, here we characterized their biophysical properties and evaluated their functionality in transport assays.

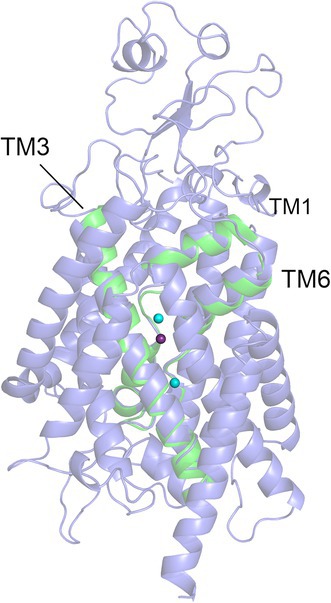

**Abbreviations**
4.1N: neuronal protein 4.1
AE1: anion exchanger 1
CCC: cation‐chloride cotransporter
CFP: cyan fluorescent protein
CHS: cholesteryl hemisuccinate
cryo‐EM: cryo‐electron microscopy
CTD: C‐terminal domain
DDM: *n*‐dodecyl‐β‐d‐maltoside
DIDS: 4,4′‐diisothiocyano‐2,2′‐stilbenedisulfonic acid
DMEM: Dulbecco's modified Eagle's medium
GABA_A_
: gamma‐aminobutyric acid type A
HEK: human embryonic kidney
KCC: K^+^‐Cl^−^ cotransporter
MOI: multiplicity of infection
nanoDSF: nano differential scanning fluorimetry
Ni‐NTA: nickel‐nitrilotriacetic acid
NKCC: Na^+^‐K^+^‐Cl^−^ cotransporter
NMDA: *N*‐methyl‐d‐aspartate
NTD: N‐terminal domain
PACSIN1: protein kinase C and casein kinase substrate in neurons 1
PAM: protein associated with Myc
SEC‐MALLS: size‐exclusion chromatography‐coupled multi‐angle static laser light scattering
TM: transmembrane
YFP: yellow fluorescent protein
β‐PIX: p21‐activated kinase‐interacting exchange factor beta

Members of the cation‐chloride cotransporter (CCC) family are secondary active ion symporters, which cotransport cations (Na^+^ and/or K^+^) and Cl^−^ [1, 2]. CCCs have crucial roles in shaping proper inhibitory signaling and neuronal connectivity in the vertebrate brain [3]. K^+^‐Cl^−^ cotransporters (KCCs) are a subfamily of CCCs that carry out the symport of K^+^ and Cl^−^ ions across the plasma membrane, whereas Na^+^‐K^+^‐Cl^−^ cotransporters (NKCCs), another subfamily of CCCs, function as symporters of Na^+^, K^+^, and Cl^−^ ions. The KCC proteins are involved in various physiological processes, such as cell volume regulation, transepithelial ion transport, neuroendocrine signaling, synapse formation, signal transmission, and blood pressure regulation [1].

Among KCCs, which include KCC1‐4 in most vertebrates [4], in particular KCC2 has gained attention because of its unique and crucial functions in the central nervous system neuronal network [5, 6]. The KCC2 protein is known to be responsible for maintaining low intracellular Cl^−^ concentration at the postsynaptic membrane, which is indispensable for inhibitory signaling by the GABA_A_ receptor [7]. It is also involved in the development of dendritic spines on excitatory glutamatergic synapses through the regulatory domains [8]. The involvement of KCC2 both in excitatory and inhibitory synapses indicates its importance as one of the key molecules for proper signal transmission in the brain. Loss of activity of this transporter has been associated with several neurological disorders including schizophrenia [9], epilepsy [10], traumatic brain injury [11], and chronic pain [12]. KCC2 is considered to be one of the drug targets toward treatment of these diseases [13, 14].

KCC proteins consist of a 12‐transmembrane α‐helix (TM) domain and N‐ and C‐terminal cytoplasmic domains (NTD and CTD). The TM domain is the actual ion transport domain, while NTD and CTD are involved in the regulation of transport activity, for example, through phosphorylation/dephosphorylation of several residues in the domains [6, 15]. CTD is also known to act as a binding scaffold to other proteins. Interactions of KCC2 CTD with protein associated with Myc (PAM) [16] and PACSIN1 [17] regulate KCC2 expression positively and negatively, respectively, and affect KCC2‐mediated ion flux accordingly, while its interactions with 4.1N [8] and β‐PIX [18] are critical for proper dendritic spine formation. Recently, cryo‐EM structures of different KCCs have been solved, showing an inward‐open conformation of the TM domain in each structure [19, 20, 21, 22, 23, 24], and in one case an outward‐open state due to the binding of an inhibitor [25]. These structures also revealed binding sites for K^+^ and Cl^−^ with highly conserved residues coordinated to those ions.

KCC activity has been analyzed by measuring changes in intra‐ or extracellular ion concentration [6]. Early work on CCC transporter activity was done with flame photometry [26]. More recently, Tl^+^ and ^86^Rb^+^ ions have often been used as substitutes for K^+^ to detect KCC activity. This is done by measuring the fluorescence of Tl^+^‐sensitive dye loaded into cells [27] or the radioactivity of ^86^Rb^+^ transported into cells [28, 29], respectively, while NH_4_^+^ has also been employed as a K^+^ surrogate to measure KCC activity [30]. On the other hand, the measurement of intracellular Cl^−^ concentration in response to KCC activity has also been done with variable methods including gramicidin perforated patch clamp recording [31], single GABA_A_ channel and NMDA channel recording [32], soma‐to‐dendrite Cl^−^ gradient [33], quinolinium halide‐sensitive indicators [34, 35], and genetically encoded Cl^−^‐sensitive indicators [36, 37]. Each of these methods to measure KCC ion transport activity has its advantages and limitations, and therefore, it will still be of great benefit to develop alternative methods to detect KCC activity.

To date, only a limited number of studies of invertebrate KCCs have been done. Among the invertebrate proteins, *Drosophila melanogaster* KCC (*Dm*KCC) has been studied the most and is suggested to be critical for proper neuronal transmission in *Drosophila* [38, 39]. Based on the evolutionary analysis of KCCs, the KCCs differentiated into different isoforms at the base of the vertebrates [4], and therefore, *Dm*KCC is phylogenetically not specifically homologous to any isoform of mammalian KCCs, although it can be inhibited by the mammalian KCC2‐specific inhibitor, VU0463271 [40, 41]. Cnidarian *Hydra vulgaris* is another species that was shown to have a protein mediating Na^+^‐independent K^+^(Tl^+^) and Cl^−^ transport (*Hv*KCC) [42]. Although the ion transport activity of *Hv*KCC appears to be significantly lower than *Rattus norvegicus* KCC2 (rat‐KCC2), comparative functional, biophysical, and structural analyses of this and the *Dm*KCC protein with KCCs from other species would be significant to understand the mechanism of ion transport and its regulation by the KCC transporter family.

In this study, we considered *Dm*KCC and *Hv*KCC as model systems and studied their structural and functional properties. The obtained biochemical and biophysical data of purified KCCs, along with molecular modeling, show common features between invertebrate and mammalian KCCs. Using recombinant *Dm*KCC and *Hv*KCC, we tested the activity of these proteins in cells by following Cl^−^ transport with a ratiometric Cl^−^‐sensitive fluorescent probe and K^+^ transport with flame photometry. Additionally, purified *Dm*KCC from Sf9 insect cells was reconstituted, and K^+^ transport into proteoliposomes was studied using a fluorescent K^+^‐sensitive probe.

## Materials and methods

### Plasmid construction

For insect cell expression, cDNAs coding *D. melanogaster* KCC (*Dm*KCC) variant B (NCBI Reference Sequence: NM_166632.2), *H. vulgaris* KCC (*Hv*KCC; NCBI Reference Sequence: XP_012555566.1), and C‐terminal domain‐truncated *Hv*KCC (*Hv*KCC‐ΔCTD) were PCR‐amplified with primers containing N‐terminal 8 × His and FLAG tags and inserted into the pFastBac1 vector, separately. For mammalian cell expression, cDNAs coding *Dm*KCC, *Hv*KCC, and rat‐KCC2b were PCR‐amplified with primers containing N‐terminal FLAG tag and inserted into the pcDNA3.1(−) vector, separately. The constructs were confirmed with DNA sequencing.

### Baculovirus generation and protein expression

The pFastBac1 vectors containing *Dm*KCC, *Hv*KCC, or *Hv*KCC‐ΔCTD genes were transformed into *Escherichia coli* DH10Bac cells, and the bacmids and baculoviruses were produced using the Bac‐to‐Bac system (Thermo Fisher Scientific, Waltham, MA, USA). Briefly, Sf9 cells on a six‐well plate were transfected with the bacmid DNA using TransIT LT1 (Mirus Bio LLC, Madison, WI, USA). After incubation at 27 °C for 5–6 days, a low‐titer virus stock was obtained. A high‐titer virus stock was obtained by infecting Sf9 cells with the low‐titer stock and shaking at 27 °C until the cell viability went below 50%. For the potassium transport activity assay, Sf9 cells at 1.6 × 10^6^ cells·mL^−1^ were infected with the high‐titer virus stock with a multiplicity of infection (MOI) of 2. At 60 h post‐infection, the cells were diluted with fresh culture medium to 2.0 × 10^6^ cells·mL^−1^ and used for the assay. For detergent solubilization screening and protein purification, Sf9 cells at 1.5–2.0 × 10^6^ cells·mL^−1^ were infected with the high‐titer virus stock with an MOI of 2. At 60 h post‐infection, the cells were harvested by centrifugation at 7000 ***g*** for 10 min.

### Detergent solubilization screening

A cell pellet of infected Sf9 cells was resuspended in 10 mm Tris–HCl pH 7.5, containing Pierce Protease Inhibitor Mini Tablets (Thermo Fisher). The cells were homogenized using a dounce homogenizer with 40 strokes. The cell lysate was centrifuged at 6000 ***g*** for 10 min at 4 °C to pellet nuclei and unbroken cells. The supernatant was centrifuged at 150 000 ***g*** for 0.5 h at 4 °C. The resulting pellet was resuspended in PBS plus 100 mm NaCl containing Pierce Protease Inhibitor Mini Tablets (Thermo Fisher). This suspension was then split into aliquots, and each stock detergent solution was added at a 1 : 10 v/v ratio to each aliquot so that each sample contained the desired final concentration of each detergent. For a positive control, milliQ was added instead of a detergent solution to an aliquot (total membrane fraction). The divided samples were then rocked at 4 °C for 16 h. The samples were then centrifuged at 150 000 ***g*** for 0.5 h at 4 °C. The resulting supernatants, detergent‐soluble fractions, were loaded on an SDS/PAGE gel. For the positive control (total membrane fraction), the centrifugation step was skipped, and the suspension was loaded on the gel. Proteins were subsequently transferred to PVDF membranes, blocked with a 5% milk solution, and probed with a mouse anti‐FLAG antibody followed by a goat anti‐mouse IgG‐HRP (horseradish peroxidase)‐conjugated antibody. The membrane was incubated with ECL Prime Western Blotting Detection Reagent (GE Healthcare, Marlborough, MA, USA), and then protein bands were detected using ChemiDoc XRS+ (Bio‐Rad, Hercules, CA, USA).

### Protein purification

#### Protein purification for oligomerization state and thermostability analyses

Cell pellets expressing KCC were resuspended in Lysis buffer (10 mm Tris–HCl pH 7.5, 5 mm MgCl_2_, 10 mm KCl, 0.001 mg·mL^−1^ DNase I, Pierce Protease Inhibitor Mini Tablets; Thermo Fisher) and then centrifuged at 200 000 ***g*** for 20 min. The pellet was resuspended in Lysis buffer, dounce‐homogenized, and then centrifuged at 200 000 ***g*** for 20 min. This step was repeated one more time. The pellet was then resuspended in High‐salt buffer (10 mm Tris–HCl pH 7.5, 5 mm MgCl_2_, 10 mm KCl, and 1 m NaCl), dounce‐homogenized, and centrifuged at 200 000 ***g*** for 20 min. This step was repeated three more times. The resultant pellet was resuspended in Solubilization buffer (50 mm Na phosphate pH 7.5, 150 mm NaCl, 50 mm KCl, 10% glycerol) and dounce‐homogenized, and 10%/2% *n*‐dodecyl‐β‐d‐maltoside (DDM)/cholesteryl hemisuccinate (CHS) stock solution was added to the sample so that the final concentration was 1%/0.2% DDM/CHS. The sample was rocked for 16 h at 4 °C and centrifuged at 170 000 ***g*** for 15 min. The supernatant was incubated with Ni‐NTA resin for 3 h at 4 °C in the presence of 10 mm imidazole. The resin was loaded onto a column and washed with Wash buffer (50 mm Na phosphate pH 7.5, 150 mm NaCl, 50 mm KCl, 10% glycerol, 0.02% DDM, 0.004% CHS, and 30 mm imidazole). The KCC protein was eluted with Elution buffer (50 mm Na phosphate pH 7.5, 150 mm NaCl, 50 mm KCl, 10% glycerol, 0.02% DDM, 0.004% CHS, 250 mm imidazole). The eluate was concentrated to 0.4–0.5 mL using an Amicon Ultra spin concentrator, 100 kDa cutoff (Merck Millipore, Darmstadt, Germany), and run through a Superdex 200 10/300 column (GE Healthcare) with Running buffer (20 mm HEPES pH 7.4, 150 mm NaCl, 50 mm KCl, 0.015% DDM, 0.003% CHS, 5% glycerol). Peak fractions were pooled and concentrated to 1.0 mg·mL^−1^.

#### *Dm*KCC purification for reconstitution to proteoliposomes

Sf9 cells from 0.5 L culture were pelleted and suspended in 0.5 L of wash buffer containing 10 mm MES/NaOH pH 6.2, 100 mm NaNO_3_, 40 mm KCl, and 1 mm MgSO_4_ and incubated with agitation at RT for 30 min. Washed cells were pelleted and resuspended in 18 mL of lysis buffer containing 5 mm Tris pH 7.5, 0.5 mm EDTA, Pierce Protease Inhibitor Mini Tablets (Thermo Fisher, 1 tablet per 50 mL). Directly before breaking cells, 0.5 mm PMSF was added. Cells were disrupted by sonication. The cell lysate was centrifuged at 2000 ***g*** for 10 min, the supernatant was collected, and the pellet was resuspended, homogenized again using a dounce‐homogenizer, and centrifuged at 2000 ***g*** for 10 min. The supernatants were combined and centrifuged at 100 000 ***g*** for 30 min. The supernatant was discarded, and the membrane pellet was suspended in 20 mL of the buffer containing 20 mm HEPES/BTP pH 7.0 and 2 mm MgCl_2_, pelleted again at 150 000 ***g*** for 30 min and finally resuspended in the same buffer. Obtained membranes were flash‐frozen and stored at −75 °C. Membranes in 2–4 mL volume were suspended in 14–16 mL of basic buffer (BB) containing 50 mm HEPES/BTP pH 7.5, 150 mm NaCl and 10% glycerol. For solubilization, DDM and CHS were added to the final concentrations of 1% and 0.005%, respectively. Suspension was stirred at 4 °C for 30 min and then clarified by centrifugation at 145 000 ***g*** for 30 min. Imidazole was added to the supernatant at the final concentration of 10 mm, and the sample was mixed with 1 mL Ni‐NTA resin and incubated using gentle agitation at 4 °C for 1 h. The resin with the bound KCC was washed with 15 mL BB containing 30 mm imidazole, 0.025% DDM, and 0.005% CHS. Then KCC was eluted with 5.5–6.0 mL BB containing 250 mm imidazole, 0.025% DDM, and 0.005% CHS. The eluted protein was concentrated using a Vivaspin 100KDa cutoff concentrator (Sartorius, Göttigen, Germany) to 100 μL (6–8 mg·mL^−1^), flash‐frozen, and stored at −75 °C in 2–3 aliquots.

### Analytical size exclusion chromatography

Size‐exclusion chromatography‐coupled multi‐angle static laser light scattering (SEC‐MALLS) was used for characterizing the oligomerization state of the purified protein. The protein sample at 1.0 mg·mL^−1^ was run through a Superdex 200 10/300 column (GE Healthcare) with running buffer (20 mm HEPES pH 7.4, 150 mm NaCl, 50 mm KCl, 0.015% DDM, 0.003% CHS, 5% glycerol) at a flow rate of 0.3 mL·min^−1^ with an HPLC system (Shimadzu, Kyoto, Japan), a MiniDAWN TREOS light scattering detector, and an Optilab rEX refractive index detector (Wyatt Technology Corp.). Data were then analyzed with the protein conjugate program in the astra 6 software (Wyatt Technology Corp., Goleta, CA, USA).

### Thermostability analysis

Protein thermostability was analyzed by label‐free nano differential scanning fluorimetry (nanoDSF, Prometheus, Nanotemper). The capillaries were filled with 15 μL of 0.5 mg·mL^−1^ *Dm*KCC or *Hv*KCC in 15 mm HEPES pH 7.4, 75 mm NaCl, 100 mm KCl, 0.015% DDM, 0.003% CHS, 5% glycerol, and placed on the sample holder. A temperature gradient of 0.5 °C·min^−1^ from 15 °C to 80 °C was applied, and the intrinsic protein fluorescence at 330 and 350 nm was recorded.

### Protein structure modeling

The model for *Dm*KCC was generated using the SwissModel server [43]. Several models were generated with various KCC structure templates. A model generated with KCC3 dimer structure (PDB ID: 6M1Y) as a template was chosen because KCC3 has the highest sequence identity to *Dm*KCC (60% identity in the modeled parts). The template structure had the bound K^+^ and Cl^−^ ions in the structure, and no other inhibitors, ligands, or mutations that could potentially affect the conformation are present. Other template structures could have also been used, as differences with other templates are rather small. The GMQE and QMEAN scores for the model were 0.64 and −4.07, respectively.

### Culture and transfection of HEK 293 cells

Human embryonic kidney (HEK) 293 cells were maintained in Dulbecco's modified Eagle's medium (DMEM), supplemented with 10% fetal bovine serum and 50 IU·mL^−1^ penicillin–streptomycin. Cells were transfected with the appropriate pcDNAs using Lipofectamine reagent 2000 (Life Technologies, Carlsbad, CA, USA) and used 40–44 h after transfection. Briefly, the cells were placed in suspension in Opti‐MEM media (6 × 10^5^ cells·mL^−1^) and mixed with 150 μL of Lipofectamine/DNA complex. Lipofectamine/DNA complex was obtained by incubating 75 μL of Opti‐MEM media with 3.5 μL of Lipofectamine reagent 2000 (mix A containing Lipofectamine 2000). The mix of 75 μL of Opti‐MEM and 0.75 μg of DNAs encoding constructs of interest (e.g., 0.5 μg of rat‐KCC2 as a positive control, mock‐KCC2 (empty vector) as a negative control, *Dm*KCC, or *Hv*KCC and 0.25 μg of SuperClomeleon; mix B containing DNA) was further prepared. Mixes A and B were mixed, incubated for 20 min at room temperature, and then incubated with freshly prepared HEK 293 cell suspension. The cells were distributed into a 96‐well plate and incubated at 37 °C, 5% CO_2_. Then, after 12 h, transfection was terminated by substitution of 90% of the Opti‐MEM media with fresh DMEM media. Cells were used in the experiments 40–44 h after transfection.

### KCC chloride transport activity assay in HEK 293 cells

To determine the activity of the rat‐KCC2, *Dm*KCC, and *Hv*KCC, the change in the fluorescence emitted by the Cl^−^ sensitive SuperClomeleon probe [44] in response to changes in [Cl^−^]_i_ concentration was recorded. HEK 293 cells expressing rat‐KCC2, *Dm*KCC, *Hv*KCC, or the mock‐KCC2 vector were loaded with [Cl^−^]_i_ by incubating them in a solution containing high [K^+^]_o_ and [Cl^−^]_o_. This solution, named “75 K^+^ solution” (containing 148 mm Cl^−^ and 140 mm K^+^ (in mm): 140 KCl, 10 HEPES, 20 d‐glucose, 2 CaCl_2_, 2 MgCl_2_, pH 7.4, osmolarity adjusted to 300 mOsm by adding Na‐gluconate), was diluted in HEPES‐buffered (HBS) solution (in mm) (140 NaCl, 2.5 KCl, 10 HEPES, 20 d‐glucose, 2.0 CaCl_2_, 2.0 MgCl_2_, pH 7.4, osmolarity 300 mOsm, adjusted using NaCl). After 10 min of loading, a K^+^‐free solution (containing 148 mm Cl^−^ and 0 mm K^+^ (in mm): 140 NaCl, 10 HEPES, 20 d‐glucose, 2 CaCl_2_, 2 MgCl_2_, pH 7.4, osmolarity adjusted to 300 mOsm by adding Na‐gluconate) was added to the extracellular media. The addition of the ^+^K‐free solution changes the sum of electrochemical gradients and provokes KCC‐dependent extrusion of Cl^−^ and changes in [Cl^−^]_i_. This triggers modification of the SuperClomeleon fluorescence and a decrease in the ratio of CFP/YFP signal (R_CFP/YFP_), which reflects the functionality of the studied KCCs. The fluorescence was measured using a microplate reader (FluostarOptima; BMG Labtech, Ortenberg, Germany) with two filter sets activated consecutively. The first measurement was performed using the filter set detecting Cl^−^ sensitive fluorescence of the YFP part of the SuperClomeleon probe (excitation 500 nm, emission 560 nm), and the second measurement was performed using the filter set detecting Cl^−^ insensitive fluorescence of the CFP part of the SuperClomeleon probe (excitation 450 nm, emission 480 nm). The intervals between measurements were 90 s. Nontransfected cells were used as a control for background fluorescence levels. Each experiment was made in triplicate. The results were analyzed using Excel and graphpad prism (GraphPad, Boston, MA, USA).

### *Dm*KCC potassium transport activity assay in Sf9 cells

Sf9 cells infected with baculovirus expressing *Dm*KCC and *Hv*KCC‐ΔCTD were used. One milliliter of the cell suspension (2.0 × 10^6^ cells·mL^−1^) was centrifuged at 500 ***g*** for 3.5 min, and then the cells were resuspended in 1 mL of the assay buffer (200 mm MES/Bis‐tris propane (BTP) pH 6.2, 2 mm MgSO_4_). The reaction was initiated by the addition of RbCl or RbNO_3_ as indicated, and after incubation for different times at 20 °C, it was stopped by centrifugation at 4 °C. Then the cells were washed with 1 mL of 500 mm ice‐cold mannitol and centrifuged at 700 ***g*** for 3.5 min. The cells pellet was resuspended in 2 mL of Li‐test solution (Internal standard Li 3000 mmol; Instrumentation Laboratory, Bedford, MA, USA) diluted 200 times with water, and K^+^ content was measured with a flame photometer (PFM 234; Instrumentation Laboratory) immediately. For studying [Cl^−^] dependence, the medium was supplemented with *N*‐methyl‐d‐glucamine chloride salt at different concentrations.

### *Dm*KCC reconstitution to proteoliposomes

Lipid/detergent binary micelles were prepared by sonication of 18 mg·mL^−1^ azolectin in 50 mm HEPES/BTP pH 7.0, 0.5% DDM, and 0.005% CHS. Approximately 0.15 mg KCC was added to 250 microL and left under gentle agitation for 20 min for ternary micelles formation. Proteoliposomes were formed after the addition of 170 μL of BioBeads (Bio‐Rad) per 250 μL of ternary micelles and gentle agitation at RT for 2 h. Protein‐free liposomes were prepared in the same way. A detailed description of the protein preparation is given in the Supporting Information.

### Potassium transport monitoring in proteoliposomes

Prepared proteoliposomes were loaded with 0.3 mm ION Potassium Green‐2 TMA+ Salt, K^+^ indicator (#ab142807; Abcam, Waltham, MA, USA) (FKG), a fluorescent K^+^‐sensitive green probe, by mild sonication. This probe has low affinity for K^+^ (Fig. S2), which requires it to be used at rather high potassium salt concentrations. The probe outside the liposomes was removed by two sequential runs through Micro Bio‐Spin P‐6 Gel Columns (Bio‐Rad) equilibrated with 50 mm HEPES/BTP pH 7.0. The K^+^ influx into proteoliposomes in the same buffer was initiated by the addition of K^+^ salts, and fluorescent changes were followed using a Hitachi F‐7000 (Hitachi, Tokyo, Japan) fluorescence spectrophotometer at λ_ex_ = 526 nm and λ_em_ = 546 nm. The sensitivity of the K^+^ influx to 4,4′‐diisothiocyano‐2,2′‐stilbenedisulfonic acid (DIDS) was tested by measuring the FKG fluorescence level at 300 s after the addition of 75 mm KCl to proteoliposomes normalized to the fluorescence level in the absence of DIDS.

## Results

### Purification and biophysical characterization of *Hv*KCC and *Dm*KCC

**Fig. 1:**
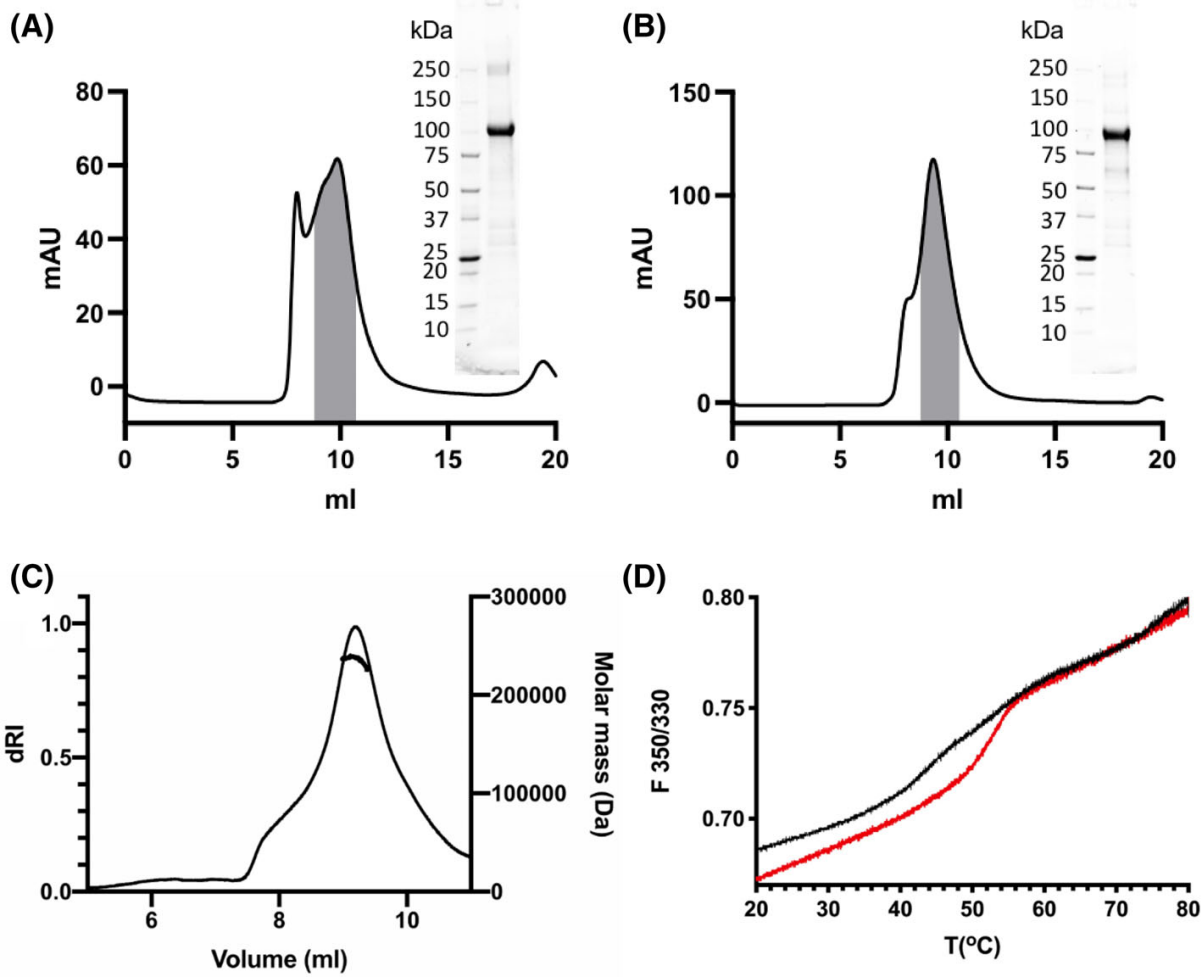
Purification and biophysical characterization of KCCs. (A) *Dm*KCC and (B) *Hv*KCC gel filtration chromatography profile with collected fractions marked in gray; insert shows the SDS/PAGE analysis of the purified material. (C) SEC‐MALLS analysis of *Hv*KCC showing an apparent dimeric species as the prominent oligomerization state with protein molecular mass of ca. 236 kDa (single measurement, with bovine serum albumin control at 1 mg·mL^−1^). (D) Differential scanning fluorimetry analysis of the thermostability of *Dm*KCC (red) and *Hv*KCC (black) measured as function of intrinsic tryptophan fluorescence emission (*n* = 1).

Both of the *Hv*KCC and *Dm*KCC constructs were expressed with the baculovirus system from *Spodoptera frugiperda* Sf9 cells. Based on detergent screening, DDM was selected to solubilize the proteins for purification (Fig. S1A,B). Both proteins were purified by Ni‐affinity chromatography followed by size exclusion chromatography, and fractions were pooled for the peak representing the presumed dimer (Fig. 1A,B). The purity of the pooled fractions was verified by SDS/PAGE. SEC‐MALLS data showed that *Hv*KCC purified with DDM in combination with CHS was in the dimeric state with a protein molecular weight of 236 kDa and a total protein/DDM/CHS complex mass of 456 kDa (Fig. 1C), which is thought to be a functional form for mammalian KCCs [3], while for *Dm*KCC, it was not possible to get accurate measurements due to aggregate carryover in the light scattering signal. However, based on elution profiles for both proteins in Fig. 1A,B, the purified main peak for *Dm*KCC corresponds to a dimer as verified for *Hv*KCC. Protein thermostability was analyzed by label‐free nanoDSF. *Hv*KCC was less stable, while *Dm*KCC had a clear transition and was also more stable with a *T*_m_ of approximately 52 °C compared to 44 °C for *Hv*KCC as measured from the data (Fig. 1D).

### Structural and sequence comparison to known KCC structures and implication for ion binding and specificity

**Fig. 2:**
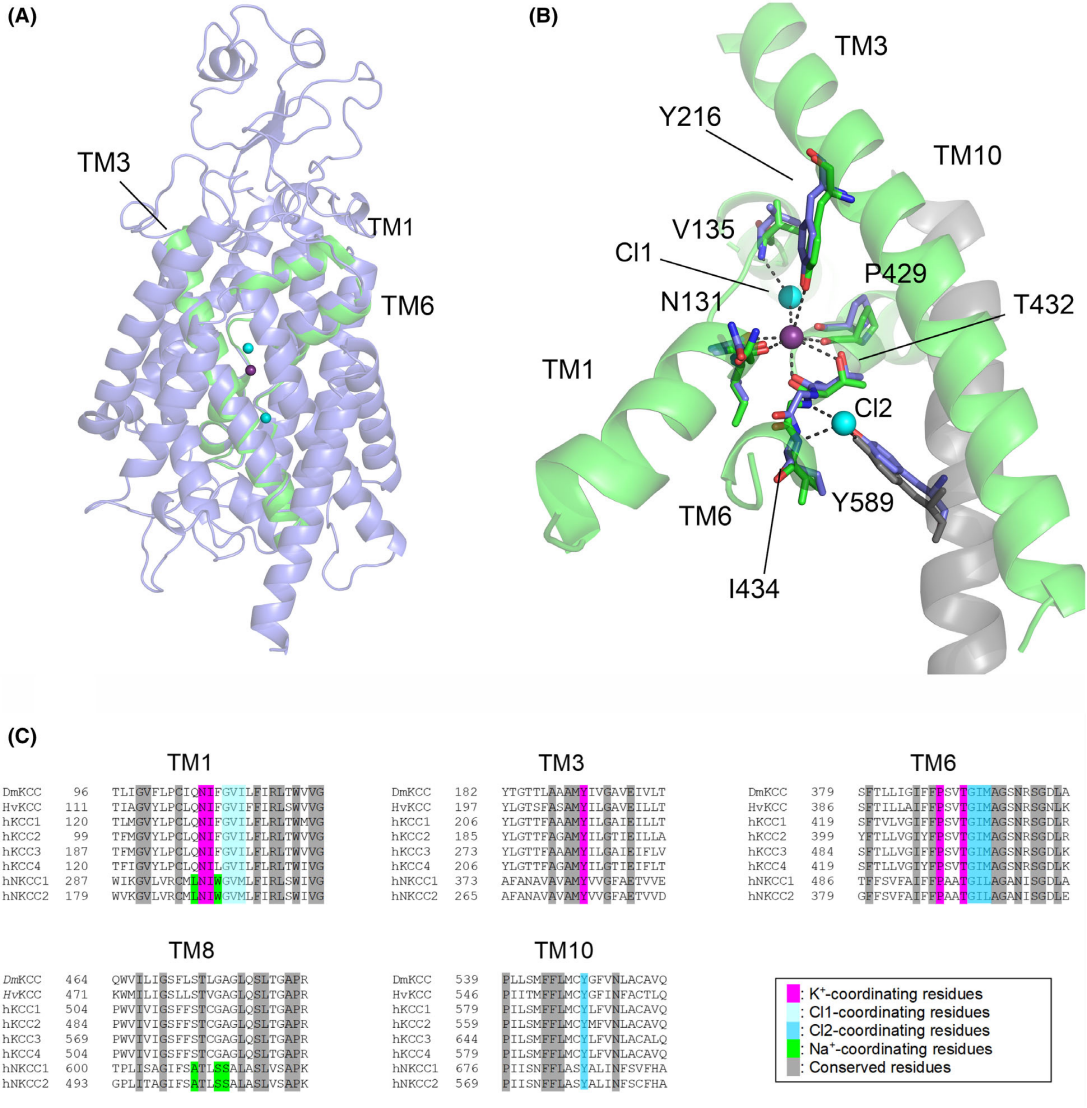
Structural model of *Dm*KCC. (A) Overall transmembrane (TM) region structure of *Dm*KCC (blue) based on homology modeling compared with the KCC4 (PDB: 7D99) ion binding helices (in green) with bound Cl^−^ (cyan) and K^+^ (magenta) ions shown as spheres. Swiss‐Model (https://swissmodel.expasy.org/) with default settings was used for homology model building. (B) The ion binding site and key residues in TM helices 1, 3, and 6 (blue), overlayed with analogous KCC4 residues (green) (KCC4 residue numbering shown, see text). KCC4 TM helix 10 is shown for context (gray) and with one contributing Tyr589 and *Dm*KCC equivalent residue conserved (blue), “Cl1” and “Cl2” Cl‐ion binding sites are indicated separately. (C) Sequence alignment of *Hv*KCC and *Dm*KCC with human KCC1‐4 (hKCC1‐4) and human NKCC1‐2 (hKNCC1‐2), with the conserved residues in TM helices 1, 3, 6, 8, and 10 colored as indicated. clustal omega (https://www.ebi.ac.uk/jdispatcher/msa/clustalo) with default parameters was used to generate the sequence alignment.

Based on the sequence alignment, *Hv*KCC has 51–53% sequence identity to human KCC1‐4 and *Dm*KCC, while *Dm*KCC has 55–58% identity to human KCC1‐4 and 52% sequence identity to *Hv*KCC. A model of *Dm*KCC was aligned with the human KCC4 structure solved at 2.9 Å resolution [22] (PDB: 7D99), which is the highest resolution dimeric wild‐type structure without inhibitors or mutations and with the ions present. Comparison of the models shows, as expected, that the overall fold is highly similar to the known structures (Fig. 2A). All the five K^+^‐coordinating, three Cl1‐coordinating, and four Cl2‐coordinating residues are highly conserved across species, including in *Dm*KCC and *Hv*KCC (Fig. 2B,C). The ion coordinating residues are equivalent to KCC4 K^+^‐coordinating (KCC4 numbering) Tyr216 and Thr432 and backbone carbonyls of Asn131 and Thr132. In the case of Cl coordination, the backbone amides of Gly134, Val135, and Ile136 (Cl1); and Gly433, Ile434, and Met345 (Cl2), and the side chain hydroxyl of Tyr589 (Cl2) coordinate the ion binding; Cl1 is additionally directly coordinated by the bound K^+^. For clarity, only selected residues are shown in Fig. 2B. The Na^+^ binding residues, as seen in the NKCC1 structure (TM1 Leu297 and Trp300, and TM8 Ala610, Ser613 and Ser614 in human NKCC1 numbering) [45] are not found in the *Dm*KCC nor *Hv*KCC model or sequence (Fig. 2B,C). Overall, the structural modeling, as demonstrated in Fig. 2 for *Dm*KCC, shows that the proteins are structurally highly conserved, and the studied proteins have the same functional sites as the mammalian KCCs.

### Activity measurements in cells expressing KCCs

**Fig. 3:**
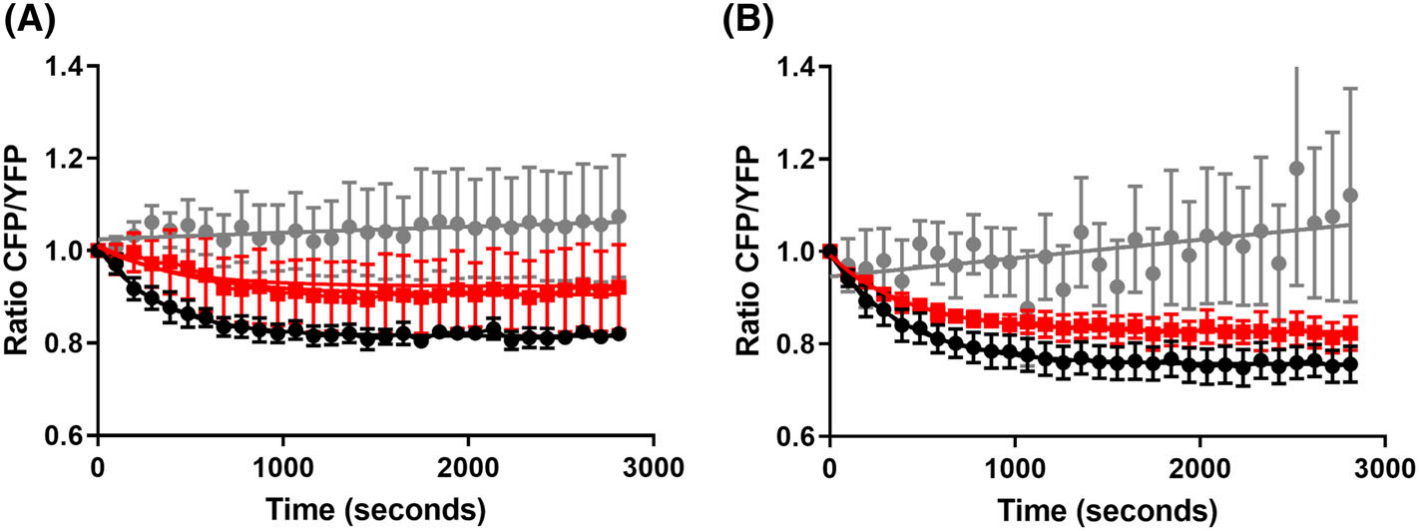
Cell‐based chloride extrusion assay for KCC activity. Cells were loaded with [Cl^−^]_i_ by incubating them in solution containing high [K^+^]_o_ and [Cl^−^]_o_. The addition of the K^+^ free solution changes the sum of electrochemical gradients and provokes KCC‐dependent extrusion of Cl^−^ and changes of [Cl^−^]_i_. This triggers modification of the SuperClomeleon fluorescence and a decrease of the ratio of CFP/YFP signal (RCFP/YFP), which reflects the functionality of the studied KCCs. Data plotted for (A) *Hv*KCC (red squares) and (B) *Dm*KCC (red squares). The wild‐type rat‐KCC2 was used as positive control in A and B (black circles). Cells transfected with the empty vector (gray circles) were used as negative control in both measurements. For each condition, results are expressed as an average of three independent experiments in triplicate, error bars indicate standard error of the mean.

*Hv*KCC was characterized functionally previously [42], and we used it here for reference toward further characterization of *Dm*KCC. We assessed the activity of both proteins using a chloride extrusion assay in cell culture. Our findings reveal that *Dm*KCC exhibits significantly higher activity, comparable to that of the rat‐KCC2 control protein for which the assay was developed. In contrast, *Hv*KCC displayed relatively low activity in the HEK 293 cell culture assay, in accordance with earlier measurements by Hartmann *et al*. [42] (Fig. 3A,B). Notably, the initial extrusion rate for the *Drosophila* protein was significantly greater, suggesting potential differences in functionality between the two proteins. Alternatively, the reduced activity of *Hv*KCC may be attributed to distinct regulatory mechanisms, such as phosphorylation or other factors, associated with the mammalian cell culture environment.

**Fig. 4:**
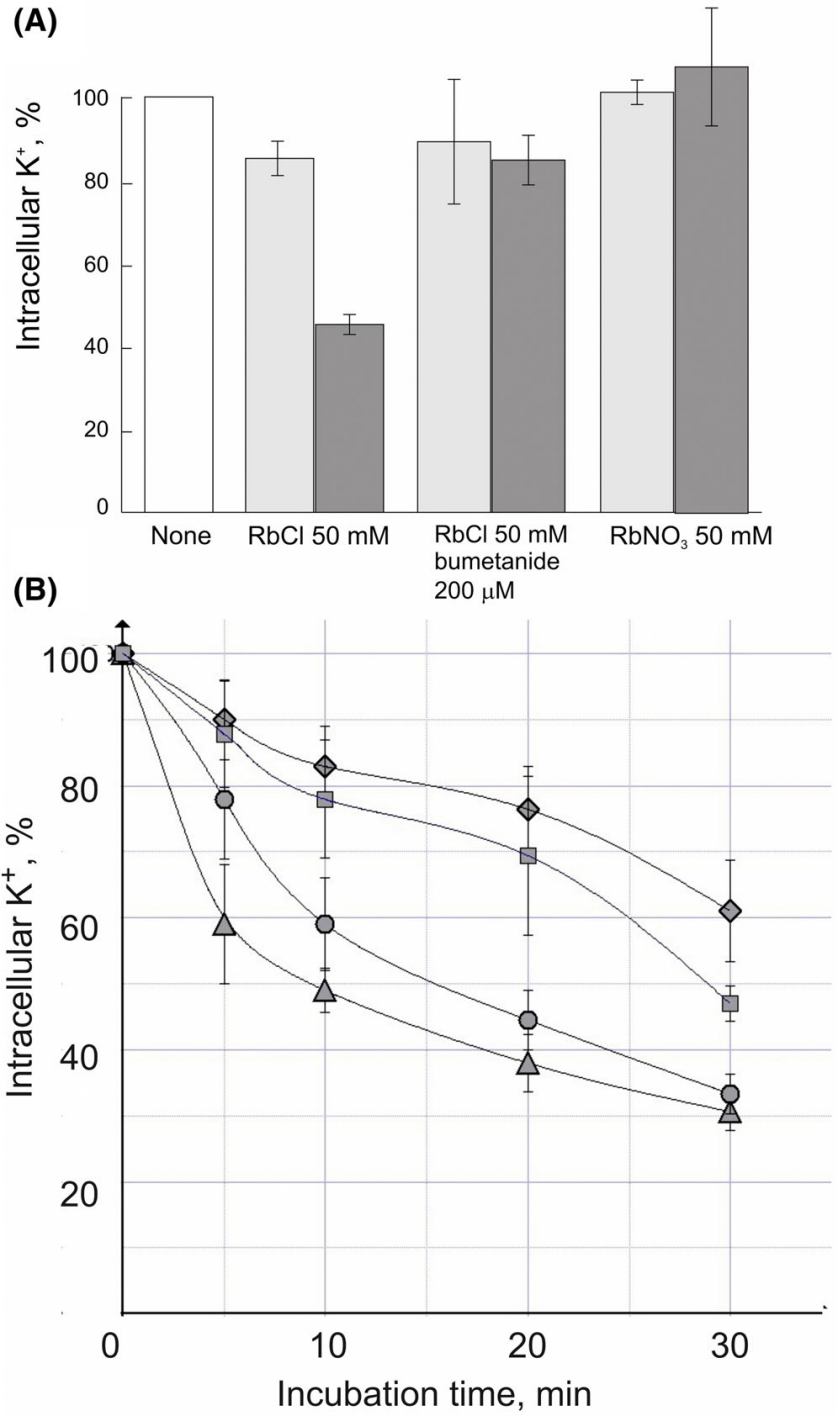
Rb^+^‐dependent K^+^ efflux by KCCs from Sf9 cells. (A) A bar chart showing *Hv*KCC‐ΔCTD (negative control) in light gray and *Dm*KCC in dark gray. K^+^ content in 20 min after Rb^+^ salts addition was normalized against its value before the addition, “none”. Standard deviation (SD) for four repeats shown. (B) Kinetics of K^+^ efflux at different [Cl^−^]: rhombs 10 mm, squares 20 mm, circles 40 mm, triangles 80 mm. At zero min, 50 mm RbNO_3_ was added. Errors are reported as SD for three repeats.

Potassium transport activity assays were performed in Sf9 cells to verify the activity of *Dm*KCC in the expression system (Fig. 4). For this purpose, RbCl or RbNO_3_ was added to the cells suspended in K^+^ and Cl^−^ or NO_3_^−^‐free medium and the decrease in the intracellular K^+^ content due to Rb^+^ influx was followed. The observed K^+^ efflux was strictly dependent on Cl^−^ (but not NO_3_^−^) and inhibited by bumetanide (Fig. 4A). Figure 4B presents transport kinetics at varying Cl^−^ concentrations, suggesting that the efflux rate increases with increasing Cl^−^ concentration.

The rather low affinity of *Dm*KCC to chloride is in line with data on mammalian KCCs: *K*_m_ = 67.3 mm for human KCC2 [27], *K*_m_ > 50 mm for rat KCC2 [29]. Rb^+^ influx was associated with active *Dm*KCC expression, since no such Rb^+^‐activated K^+^ loss was found in Sf9 cells expressing inactive CTD‐truncated *Hv*KCC. Therefore, a novel cell‐based assay for the activity of recombinant KCCs expressed in insect cells was constructed, which can be useful for future studies on ion transporters.

### Activity measurements of isolated KCC in the reconstituted system

**Fig. 5:**
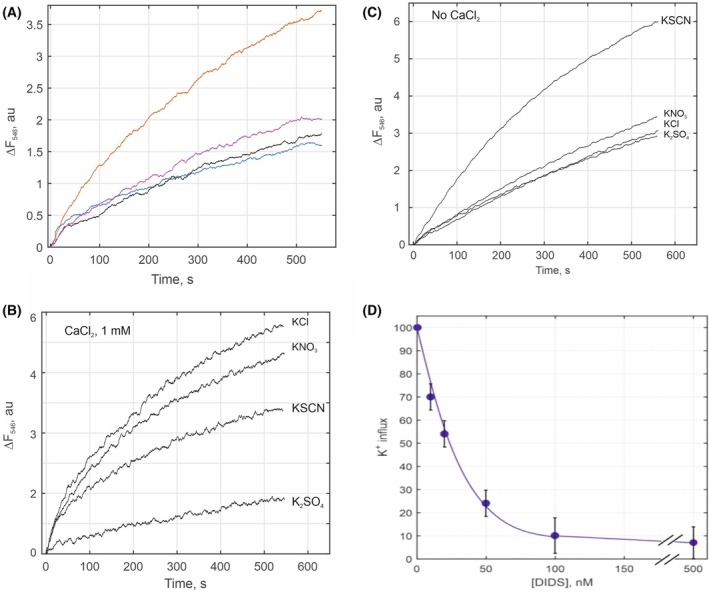
K^+^ influx into proteoliposomes mediated by reconstituted *Dm*KCC. K^+^ salts were added to proteoliposomes at zero time at concentration equivalent to 75 mm K^+^. (A) Activation of K^+^ influx initiated by KCl in the presence of different salts: 1 mm CaCl_2_ (orange), 1 mm MgSO_4_ (purple), 1 mm EDTA (black), no addition (blue); (B) Dependence of K^+^ influx on counter anions in the presence of 1 mm CaCl_2_. (C) Dependence of K^+^ influx on counter anions in the absence of CaCl_2_. (D) K^+^ influx inhibition by DIDS. Only Ca^2+^‐dependent component is sensitive to DIDS. The data of three different reconstitutions were averaged (D), error calculated as SD, and representative data shown in A–C.

Next, potassium influx into proteoliposomes with reconstituted *Dm*KCC was measured. Surprisingly, it was observed that the activity increased in the presence of Ca^2+^, which has not been observed previously with any KCCs. Potassium transport was composed of two components: Ca^2+^‐dependent and Ca^2+^‐independent (Fig. 5A–C). The ratio between these components varied from one purification of *Dm*KCC to another. Ca^2+^‐dependent component had different amplitude determined by anions present: Cl^−^ ≥ NO_3_^−^ > SCN^−^ ≫ SO_4_^2−^ (Fig. 5B), whereas anions had no effect on Ca^2+^‐independent component (Fig. 5C). No dependence on Ca^2+^ or anions on passive K^+^ influx into protein‐free liposomes was observed, except SCN^−^, well‐known for its higher permeability across membranes. Ca^2+^‐independent component includes passive potassium transport through lipid bilayer and shares its properties. No or negligible effects on K^+^ influx into proteoliposomes were observed by the addition of conventional KCC inhibitors, bumetanide and furosemide (Fig. S3). In contrast, an inhibitor of anion transporters and channels, DIDS, which is also reported as a KCC inhibitor [46], efficiently suppressed Ca^2+^‐dependent component of K^+^ influx at low concentrations (IC_50_ = 25 nm) (Fig. 5D). Ca^2+^‐independent component was insensitive to DIDS.

## Discussion

Studying the invertebrate KCCs revealed that at least *Dm*KCC clearly shares basic properties with mammalian K^+^‐Cl^−^ cotransporters. The purified *Dm*KCC tends to maintain a dimeric state like mammalian KCCs, and we showed that the ion‐binding residues are conserved based on structure prediction from closely related KCC structures recently solved experimentally. The isolated *Dm*KCC is rather thermostable, which makes the protein suitable for further studies. While recombinant *Hv*KCC has relatively weaker Cl^−^‐efflux activity (Fig. 3A), which is in line with the previously reported data [42], recombinant *Dm*KCC is functional, as observed Cl^−^ efflux from HEK 293 cells is comparable to the efflux mediated by rat‐KCC2 in our study (Fig. 3B) We used Rb^+^ as a marker for potassium transport as in [47], but we followed the changes in intracellular potassium concentration due to Rb^+^ uptake (Fig. 4). We showed that this Rb^+^ uptake is inhibited by bumetanide and depends on chloride. It is known that bumetanide is a specific inhibitor for NKCC at lower concentration but is also used to inhibit KCC at higher concentration, for example, with half maximal inhibition (*K*_0.5_) = 180 μm for rabbit KCC1 and *K*_0.5_ ~ 900 μm for mouse KCC4 [47]. It should be considered that the expression of foreign recombinant ion‐transporting proteins may lead to a cascade of events within a cell, including posttranslational modification, regulation of this protein, and/or activation of intrinsic native transport systems. Studying the transport process due to KCC expression is complicated because cells from different tissues, each with unique sets of ion‐transporting systems, may respond in diverse ways. Therefore, it is crucial to elucidate the specific mechanism of KCC function, which could be approached by isolating the protein and reconstituting it into liposomes. Previously, Zhao *et al*. [25] showed that the influx of Tl^+^ into proteoliposomes with reconstituted human KCC1 was inhibited by VU0463271, a KCC inhibitor tested on cells, but the transport dependence on Cl^−^ was not investigated. Our studies of reconstituted *Dm*KCC revealed unexpected properties of the protein: potassium influx mediated by *Dm*KCC was a combination of two components, Ca^2+^ dependent and Ca^2+^ independent, indicating two populations of the protein. This may be due to only partial functional expression and/or regulation of *Dm*KCC in Sf9 cells. It is likely that the Ca^2+^ dependent component reflects the intrinsic activity of *Dm*KCC, as it discriminates counter anions and is inhibited by an inhibitor of anion transporters and channels, DIDS. The other component, which is insensitive to DIDS, bumetanide, and furosemide and promiscuous to anions, may reflect artificial K^+^ permeability due to disturbed lipid bilayer by the presence of partly improperly folded and/or aggregated protein in the preparation.

Ca^2+^ dependence of KCCs was not previously tested, as all experiments were performed on cells in solutions containing Ca^2+^. In our study, we showed in the reconstituted proteoliposomes that K^+^ influx by *Dm*KCC was facilitated by Ca^2+^ (Fig. 5). Whether this Ca^2+^ effect is common among different species and different CCCs remains to be studied further. Data on KCCs inhibition by DIDS are scarce in the literature. At μm range, it was found to inhibit recombinant rabbit KCC1 and mouse KCC4 in oocytes [47] and Cl^−^‐dependent K^+^ efflux in sheep erythrocytes, which was thought to be due to KCC [48]. DIDS is a well‐known conventional inhibitor of anion transporters and channels including some proteins of the *SLC4* family (bicarbonate transporters) [49], *SLC16* family (monocarboxylate transporter) [50], *SLC26* family (small anion transporters) [51], ClC family (chloride channels and transporters) [52], and CaCC family (Ca^2+^‐dependent chloride channels) [53]. Considering the broad specificity of this inhibitor, it is possible that DIDS inhibits those proteins including KCCs by recognizing and binding to positively charged residues around anion‐binding sites. Recent cryo‐EM structure of AE1, an *SLC4* family protein, bound to DIDS indicates that the inhibitor probably overlaps with the anion‐binding site [54]. It should be mentioned that the activity of CaCCs [55] and some ClC [56, 57] proteins is also regulated by Ca^2+^, although whether it can be related to activation of *Dm*KCC by Ca^2+^ is unclear.

Some observed contradictions between *Dm*KCC mediated Rb^+^ influx in cells and K^+^ influx in proteoliposomes are challenging to explain. In this study, bumetanide was an efficient inhibitor in cells but not in the reconstituted system, and nitrate prevented K^+^ efflux in cells but stimulated it in proteoliposomes. This could be due to the different states of *Dm*KCC in cells and the isolated form and/or the activation of intrinsic KCC or NKCC in Sf9 cells, which transport K^+^ out of the cell in response to Rb^+^ influx mediated by *Dm*KCC.

In summary, we have characterized purified KCCs from *D. melanogaster* and *H. vulgaris* expressed from mammalian cell and insect cell culture, demonstrating that *Dm*KCC, in contrast to *Hv*KCC, has well‐detectable K^+^‐Cl^−^ transport activity. We established cell‐based and reconstituted protein assays to monitor the activity of the recombinant protein, which are valuable for ion transport protein quality control. We also showed that *Dm*KCC shares properties with mammalian KCCs, such as similarity of the ion binding residues, the dimeric state of the purified protein, and the capability to transport Cl^−^ and K^+^. Additionally, the activity of isolated *Dm*KCC was found to be Ca^2+^ dependent and inhibited by the anion transporters/channels inhibitor DIDS, which are reminiscent of some chloride transporting proteins, CaCCs, and some ClCs.

## Conflict of interest

The authors declare no conflict of interest.

## Peer review

The peer review history for this article is available at https://www.webofscience.com/api/gateway/wos/peer‐review/10.1002/2211‐5463.70063.

## Author contributions

TK, MV, and SF conceived and designed the project. SF, MV, and CDS acquired the data. All authors contributed to data analysis and interpretation. TK, SF, and MV wrote the paper. All authors contributed to the writing and editing. TK and CR supervised the work.

## Supporting information


**Fig. S1.** Detergent solubilization screening for purification of KCCs.
**Fig. S2.** Fluorescence titration of Potassium Green‐2 TMA+ Salt, K^+^ indicator (#ab142807; Abcam) (FKG) with K^+^.
**Fig. S3.** K^+^ influx into proteoliposomes mediated by reconstituted *Dm*KCC in the presence of bumetanide and furosemide or in their absence.

## Data Availability

Data are available upon request.
