# Supplementary material for: Biophysical characterization and ion transport with cell‐based and proteoliposome reconstitution assays of invertebrate K+‐Cl− cotransporters
Source: FEBS Open Bio. 2025 Jun 13;15(9):1532–45. doi: 10.1002/2211-5463.70063 (PMC12401179; doi:10.1002/2211-5463.70063)
Supplement: Supplementary file 1 — Fig. S1. Detergent solubilization screening for purification of KCCs. Fig. S2. Fluorescence titration of Potassium Green‐2 TMA+ Salt, K+ indicator (#ab142807; Abcam) (FKG) with K+. Fig. S3. K+ influx into proteoliposomes mediated by reconstituted DmKCC in the presence of bumetanide and furosemide or in their absence. [file FEB4-15-1532-s001.docx]

**Supplementary data**

# **Biophysical characterization and ion transport with cell-based and proteoliposome reconstitution assays of invertebrate K^+^-Cl^−^ co-transporters**

Satoshi Fudo, Marina Verkhovskaya, Coralie Di Scala, Claudio Rivera, Tommi Kajander


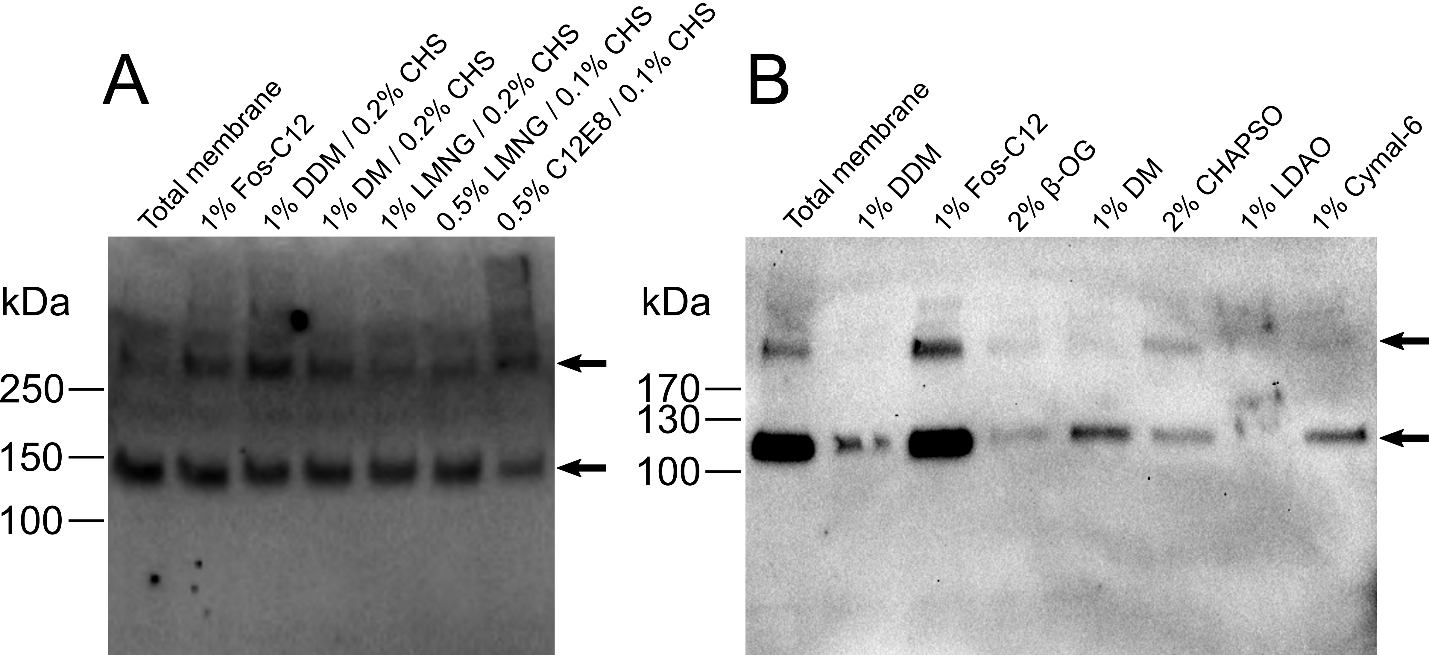


**Figure S1. Detergent solubilization screening for purification of KCCs. A**) *Dm*KCC and **B**) *Hv*KCC western blot detection from solubilization with various detergents compared to total membrane preparation.


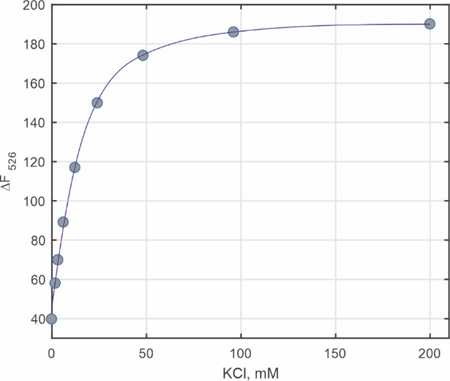


**Figure S2. Fluorescence titration of Potassium Green-2 TMA+ Salt, K+ indicator (#ab142807, Abcam) (FKG) with K^+^.** Medium: 50 mM HEPES/BTP pH 7.0. λ_ex_=526 nm and λ_em_=546 nm.


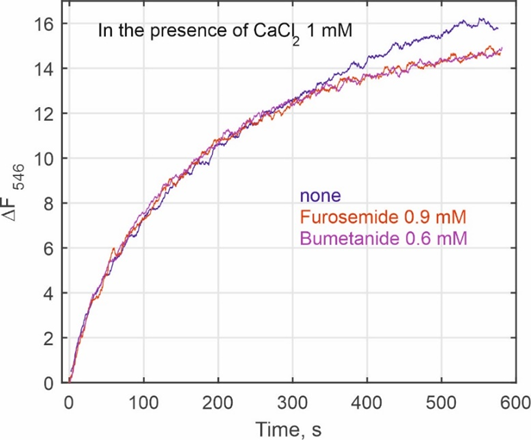


**Figure S3. K^+^ influx into proteoliposomes mediated by reconstituted *Dm*KCC in the presence of bumetanide and furosemide or in their absence.** 75 mM KCl was added at zero time.
